# Myeloid-mediated cerebral amyloid vasculitis and the potential role of the immune response in brain atrophy

**DOI:** 10.1172/JCI195137

**Published:** 2025-06-19

**Authors:** Rudy J. Castellani, Hinda Najem, Amy B. Heimberger, Pouya Jamshidi

**Affiliations:** 1 Department of Pathology and; 2 Department of Neurosurgery, Northwestern University Feinberg School of Medicine, Chicago, Illinois, USA.

**Keywords:** Immunology, Neuroscience, Vascular biology, Alzheimer disease, Immunotherapy

**To the Editor:** Anti–amyloid β (Anti-Aβ) immunotherapies have shown evidence of target engagement (1). Although most clinical trials have demonstrated no clinical benefit, recent trials of humanized monoclonal anti-Aβ antibodies have suggested a reduction in cognitive deterioration (2), albeit similar to that seen after treatment with conventional drugs (3). Furthermore, the “disease modification” might be microglial clearance of Aβ (4) rather than cognition per se. This was the case in previously reported findings from the AN-1792 trial (1). The extent of targeting of vascular Aβ (cerebral amyloid angiopathy [CAA]) and the mechanism(s) of clearance are incompletely resolved. Moreover, vascular Aβ targeting may play a role in amyloid-related imaging abnormalities (ARIA) — a common side effect of anti-Aβ therapy.

We recently reported findings in a patient who exhibited stroke-like signs after receiving lecanemab and died of multifocal intracerebral hemorrhage (5). At autopsy, Aβ plaque phagocytosis and necrotizing vasculitis were observed, indicating targeting of Aβ plaques and CAA through antibody-mediated recruitment of macrophages. The sampling details and semiquantitative analysis of CAA, vasculitis, necrotizing vasculitis, and hemorrhage have been previously reported (5). Importantly, there was no vascular territory ischemic stroke at autopsy. A long-standing hypothesis has been that Aβ might be cleared via a perivascular route with anti-Aβ therapy and that ARIA might represent a transient increase in vascular/perivascular Aβ during Aβ plaque removal. We therefore conducted additional analysis of the lecanemab-treated case alongside a case with untreated Alzheimer’s disease and severe CAA and high Alzheimer’s disease neuropathologic change, using spatial proteomic multiplex immunofluorescence (Supplemental Table 1 and Supplemental Methods; supplemental material available online with this article; https://doi.org/10.1172/JCI195137DS1). We also examined the lecanemab-treated case’s spleen for the accumulation of Aβ and GFAP, alongside the spleen from another patient with high Alzheimer’s disease neuropathologic change but no prior anti-Aβ immunotherapy.

In the lecanemab-treated case, there was an accumulation of CD11c^+^ antigen-presenting cells (APCs) surrounding the Aβ^+^ vessels (Figure 1A). These CD11c^+^ APCs expressed LAMP1 and Rab5, indicating activation and phagocytic capacity and involved CAA within the vessel wall (Figure 1B, red arrows). These findings were not observed in the untreated case. P2RY12^+^CD11c^+^ phagocytic microglia, expressing LAMP1 and Rab5, were also observed within the Aβ plaques in the lecanemab-treated case but not the untreated case (Supplemental Figure 1). There was accumulation of fibronectin and fibrinogen within the vessel walls with surrounding hemorrhage in the lecanemab-treated case, consistent with the necrotizing vasculitis with fibrinoid necrosis (Figure 1C). Rather than the Aβ being cleared via a perivascular route, the vascular pathology was more consistent with a vasculitis. Moreover, the primary cell type involved in the CAA immune response appears to be periphery-derived CD11c^+^ APCs rather than P2RY12^+^ microglia.

MRI studies of patients receiving anti-Aβ therapy have demonstrated a marked reduction in brain volume. In a systematic review, anti-Aβ immunotherapy was associated with 38.7% greater ventricular enlargement, with a significant correlation between ventricular volume and ARIA frequency (*P* = 6.22 × 10^–7^) (6). To ascertain if this volume loss was due to immune clearance of cerebral parenchyma, the spleens of the lecanemab-treated and an untreated Alzheimer’s disease case were analyzed. Aβ^+^CD68^+^ macrophages were found in the spleens of both the lecanemab-treated case and the untreated case (~60% of macrophages in both), and we believe this to be a novel finding. Moreover, glial fibrillary acidic protein^+^ (GFAP^+^) CD68^+^ macrophages (~42% of CD68^+^ cells, 327 cells counted) were found only in the lecanemab-treated case (Figure 1, D and E). In addition, myelin basic protein (MBP) was noted in 20% of splenic macrophages in the lecanemab-treated case but not in the untreated disease case. No neuronal markers (MAP2, tau [AT8]) were noted in splenic macrophages. Although the presence of GFAP^+^/CD68^+^ and MBP^+^/CD68^+^ macrophages in the spleen could be related to intracerebral hemorrhage, it raises a provocative hypothesis that brain volumetric loss may be due to clearance of brain parenchymal elements. Certainly, the findings reported here are limited by their anecdotal nature and are hypothesis generating. These findings nevertheless raise the possibilities that (a) necrotizing vasculitis might underly ARIA; (b) CAA may be cleared by a systemic immune response; and (c) brain volumetric loss may be due to immune-mediated clearance of brain parenchyma. Analysis of the frequency of splenic Aβ, GFAP, and MPB with and without treatment is warranted.

## Author contributions

RJC, ABH, and PJ provided funding and resources. RJC, HN, and PJ provided sample collection and methodology. HN analyzed data. RJC and HN wrote the first draft of the manuscript. All authors reviewed and approved the final version of the manuscript.

## Supplementary Material

Supplemental data

## Figures and Tables

**Figure 1:**
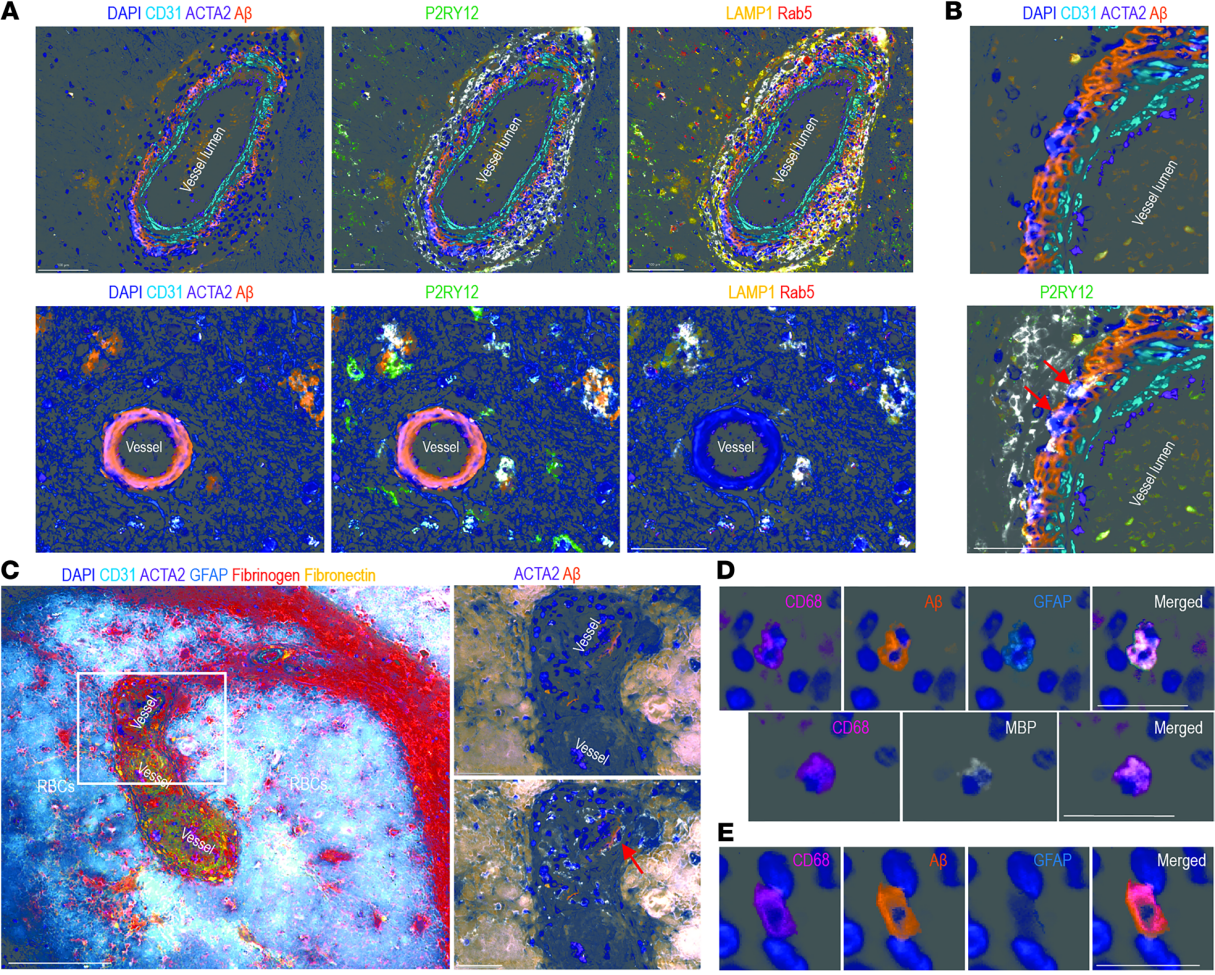
Spatial proteomic multiplex immunofluorescence analysis comparing a lecanemab-treated case with an untreated Alzheimer’s disease case. (**A**) Multiplex images showing differences in cortical vessel wall and immune infiltration between cases. CD31 was used to indicate endothelial cells; ACTA2 was used to indicate smooth muscle; CD11c was used to indicate antigen-presenting cells; P2RY12 was used to indicate microglia; LAMP1 was used to indicate activated myeloid cells; Rab5 was used to indicate phagocytosis. Aβ, amyloid β. Scale bars: 100 μm (top); 50 μm (bottom). (**B**) Multiplex images showing CD11c^+^ APCs infiltrating the cortical vessel wall with cytoplasmic Aβ (red arrows). Scale bar: 50 μm. (**C**) Multiplex images in the lecanemab-treated case (left), showing fibrinogen and fibronectin within cortical vessels along with hemorrhage. Scale bar: 200 μm. Remnants of the vessel wall and Aβ amyloid within CD11c^+^ APCs (red arrow) are shown (right). Scale bar: 50 μm. (**D** and **E**) Representative images of spleen from the lecanemab-treated or untreated case showing expression of Aβ, GFAP, or MBP within CD68^+^ monocytes. Scale bars: 20 μm. Of note, Aβ^+^ macrophages were present in the lecanemab-treated and untreated Alzheimer’s disease spleen (~60%), while GFAP^+^ and MBP^+^ splenic macrophages were noted only in the lecanemab-treated spleen (42% and 20%, respectively).

## Version 1 06/19/2025

In-Press Preview

## Version 2 09/02/2025

Electronic publication
