# Supplementary material for: Myeloid-mediated cerebral amyloid vasculitis and the potential role of the immune response in brain atrophy
Source: J Clin Invest. 2025 Jun 19;135(17):e195137. doi: 10.1172/JCI195137 (PMC12404737; doi:10.1172/JCI195137)
Supplement: Supplemental data [file jci-135-195137-s254.pdf]

Spatial proteomic multiplex immunofluorescence (IF) staining

Sequential IF was performed using Lunaphore's COMET™ system with automated staining, imaging, and elution cycles. The multiplex immunofluorescence panel included X antibodies (**Supplementary table 1**). All antibodies were validated using conventional immunohistochemistry and/or immunofluorescence (IF) staining, in conjunction with the corresponding fluorophore and the spectral 4',6-diamidino-2-pheynlindole (DAPI; ThermoFisher Scientific) counterstain. For optimal concentration and the best signal/noise ratio, all antibodies were tested at three different dilutions, starting with the manufacturer-recommended dilution (MRD), then MRD/2, and MRD/4. Secondary Alexa Fluor 555 (ThermoFisher Scientific) and Alexa Fluor 647 (ThermoFisher Scientific) were used at 1/200 and 1/400 dilutions, respectively. The optimizations and full runs of the panel were executed using the sequential IF (seqIF™) methodology integrated into the COMET™ control software (*Rivest, F., et al., Fully Automated Sequential Immunofluorescence (seqIF) for Hyperplex Spatial Proteomics. bioRxiv, 2023*). The staining is performed following automated cycles of 2 antibodies' staining at a time, followed by imaging and elution, where no human intervention is required. All reagents were diluted in Multistaining Buffer (BU06, Lunaphore Technologies). The elution step lasted 2min for each cycle and was performed with Elution Buffer (BU07-L, Lunaphore Technologies) at 37°C. Quenching lasted for 30sec and was performed with Quenching Buffer (BU08-L, Lunaphore Technologies). Imaging was performed with Imaging Buffer (BU09, Lunaphore Technologies). The incubation times were set at 4min for all primary antibodies and secondary antibodies at 2min. Imaging was performed with an integrated epifluorescent microscope at 20x magnification. Image registration was performed immediately after concluding the staining and imaging procedures by COMET™ control software. Each protocol resulted in a multi-stack OME-TIFF file where the imaging outputs from each cycle were stitched and aligned. The OME-TIFF files contain a DAPI image, intrinsic tissue autofluorescence in TRITC and Cy5 channels, and a single fluorescent layer per marker. Subsequent analysis was performed using the HORIZON Viewer™ software from Lunaphore. The background autofluorescence cycle was subtracted from each subsequent cycle using the subtraction feature integrated within the Viewer™. Each marker was then pseudo-colored and its signal thresholded for visualization and generation of snapshot Tiff files.

Supplemental Table 1: Primary and secondary antibodies used for multiplex IF staining.

MARKER	MANUFACTURER	CATALOGUE NUMBER	CLONE	DILUTION
**DAPI**	Thermofisher	62248	N/A	1/1500
**AF 555**	Thermofisher	A32727	N/A	1/200
**AF 647**	Thermofisher	A32733	N/A	1/400
**CD31**	Abcam	Ab225883	EPR17259	1/1500
**ACTA2**	Abcam	Ab7517	1A4	1/1000
**GFAP**	Sigma	MAB360	GA5	1/3000
**MAP2**	Abcam	Ab183830	EPR19691	1/100,000
**Tau**	Thermofisher	MN1020	AT8	1/1500
**β-Amyloid**	Biolegend	800701	4G8	1/1000
**P2RY12**	Atlas Antibodies	HPA014518	Polyclonal	1/1000
**CD11c**	Abcam	Ab52632	EP1347Y	1/300
**CD68**	Dako Agilent	GA613	PG-M1	No dilution required
**LAMP1**	Cell Signaling	9091S	D2D11	1/400
**RAB5**	Cell Signaling	3547S	C8B1	1/200
**Fibronectin**	Abcam	Ab2413	Polyclonal	1/1000
**Fibrinogen**	Abcam	Ab34269	Polyclonal	1/500
**MBP**	Santa Cruz	sc-271524	F-6	1/1000

N/A: not applicable.

Data availability: Underlying, deidentified data may be obtained from the corresponding author upon request.

Sex as a biological variable: This is a report of a single case (female decedent) involving a disease process that is not specific to sex and a therapeutic that is administered to male and female patients.

Statistics: This is a hypothesis-generating, qualitative study of a single case with no statistical analyses.

Study approval: This study was submitted to the Institutional review board and was determined that the research does not involve human subjects (HRP-503).

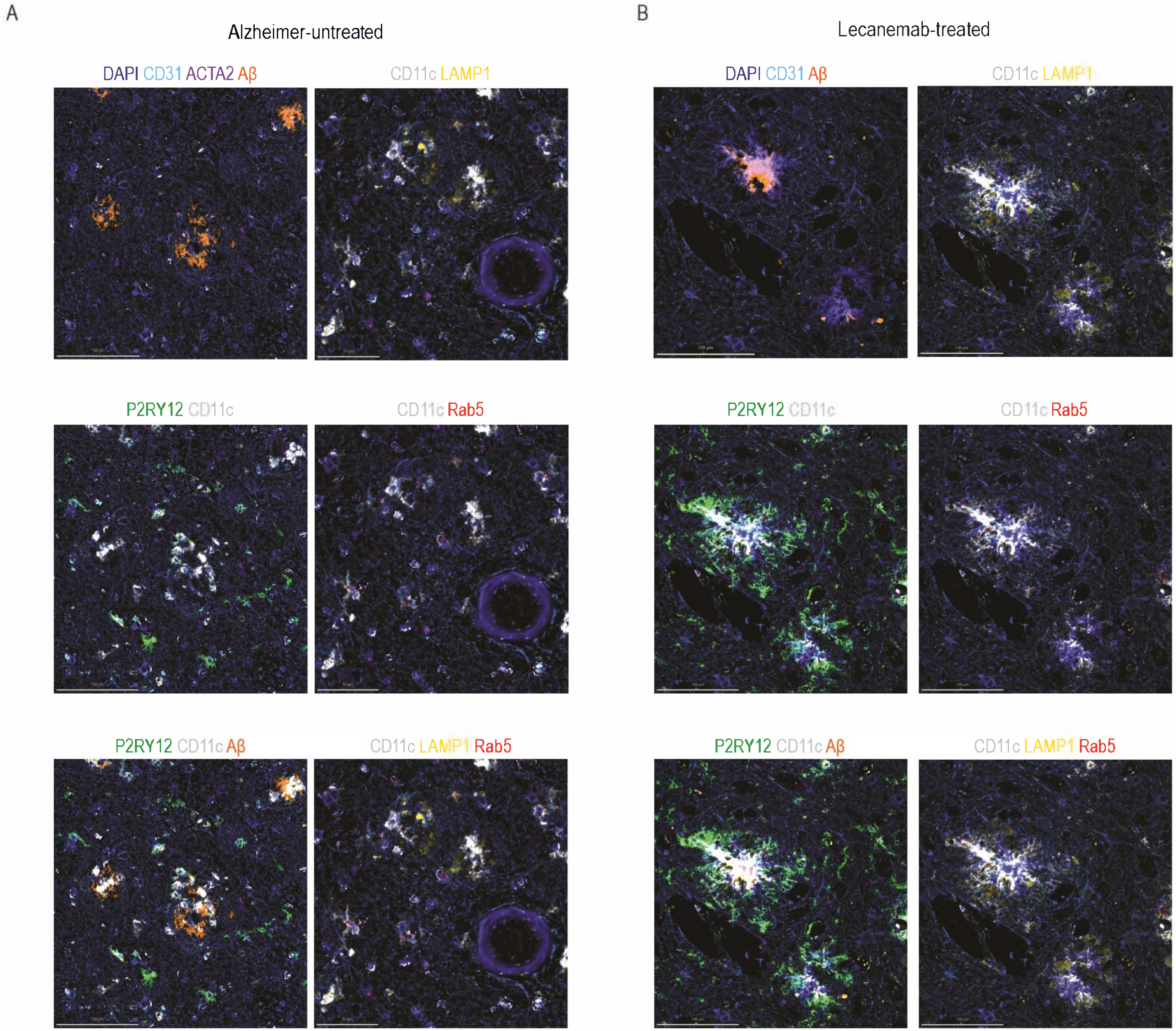

Supplemental Figure 1.
